# Detection and molecular characterization of porcine circovirus type 2 from piglets with Porcine Circovirus Associated Diseases in Colombia

**DOI:** 10.1186/1743-422X-11-143

**Published:** 2014-08-08

**Authors:** Maria Antonia Rincón Monroy, Gloria Consuelo Ramirez-Nieto, Victor Julio Vera, Jairo Jaime Correa, Jose Dario Mogollón-Galvis

**Affiliations:** Colombian Agriculture Institute - ICA, National Laboratory of Veterinary Diagnostic, Bogotá D.C, Colombia; School of Veterinary Medicine, National University of Colombia, Carrera 30 No. 45-03, Edificio 561B, Bogotá D.C, Colombia

**Keywords:** Porcine circovirus type 2 (PCV2), Porcine circovirus-associated disease (PCVAD), Genetic characterization

## Abstract

**Background:**

The porcine circovirus-associated disease (PCVAD) has been known since 1991 in Canada, but the first outbreak of PCVAD in Colombia was reported in 2007. In order to understand the molecular epidemiology of the disease and to establish the origin of the virus in the country, the study presented here intended to evaluate the presence of PCV2-associated systemic infection in piglets from different geographical regions over a period of 9-years (2002 -2010). The analysis included samples collected before, during and after outbreaks of PCVAD in pigs from Colombia. The PCV2 ORF2 from the positive samples was sequenced and used to determine the genotypes of the strains and to study the dynamic of these genotypes throughout the time.

**Results:**

PCV2 DNA was detected in cases related to PCV2-associated systemic infection as well as in healthy pigs with a presumable persistent infection. The analysis of the ORF2 nucleotide full length sequence of twenty-three strains allowed to divide them into two groups: PCV2a and PCV2b. At the amino acid level the main variations in the sequence of the capsid protein were found in regions located within the immunoreactive areas.

**Conclusions:**

The results of this study demonstrated for the first time, that the two subgroups: PCV2a and PCV2b have been circulating in swine from Colombia. In addition, the study showed that genotype PCV2b is present in Colombian pigs suffering from both clinical and presumable persistent infection and that the PCV2b genotype was present in the Colombian pig population even before recognition of the disease in the country and it became predominant through time.

## Background

Porcine circovirus type 2 (PCV2), a member of the *Circoviridae* family, is a small non-enveloped virus, containing a single-stranded circular deoxyribonucleic acid (DNA) genome [1], it is distributed worldwide and is considered to be an important emerging pathogen associated with several different syndromes and diseases in pigs, collectively grouped as porcine circovirus diseases (PCVD) [2]. PCV2 is the major infectious agent of PCVAD in pigs, a multifactorial disease, considered one of the most economically important swine diseases worldwide [3]. PCV2-associated systemic infection is clinically characterized by wasting, dyspnea, and lymphadenopathy and might be associated with diarrhea, pallor, and jaundice [4]. The most relevant histological lesions in this condition occur in lymphoid organs and consist of extensive lymphocytic depletion, macrophage infiltration, a few multinucleated giant cells, and botryoid basophilic cytoplasmic inclusion bodies [5].

PCV2 DNA genome is about 1767–1768 nucleotides long [6] and encodes for three open reading frames (ORFs) [7]. The ORF1 encodes the replication-related proteins: Rep (helicase) and Rep’ (nickase) that is the result of Rep-transcript alternative splicing process [8, 9]. The ORF2 encodes the capsid protein, the only structural viral protein that is also the most variable nucleotide sequence of the viral genome [10, 11]. Finally, the ORF3 embedded within ORF1, encodes a protein that is not essential for viral replication but is fundamental to the development of the viral pathogenesis [12, 13].

PCV2 is classified into two main genotypes, PCV2a and PCV2b which were further subdivided into different clusters, 1A–1C and 2A–2E for PCV2b and PCV2a, respectively [11]. A third genotype, PCV2c, has only been found in Denmark [14]. A new type of PCV referred to as PCV1/2a was reported in Canada in 2009, it was found to be a chimeric virus containing ORF1 of PCV1 and ORF2 of PCV2a [15]. Recently, two additional genotypes PCV2d and PCV2e, were described following sequence analysis of PCV2 isolates from China [16]. However, a subsequent analysis of data sequences failed to support the new genotypes reported [17].

In South America, PCV2 has been circulating in Brazil since 1988 [18], but it was also detected in Argentina [19, 20], and Chile [21]. PCV2-associated systemic infection was first reported in 2007 [22] in Colombia from pig farms located in different regions. In spite of that, genetic information about PCV2 strains in pig herds from the country had been unavailable until now. The main purpose of this work was to establish molecular detection and to achieve genetic and phylogenetic comparisons of full-length sequences of the ORF2 from PCV2 strains recovered from clinically healthy and PCV2-associated systemic infection affected pigs from different production systems in Colombia. Another purpose was to study the viral genotypes dynamics through time to trace temporal changes, in order to understand the molecular epidemiology of PCV2 in Colombia.

## Results

### PCV2 DNA detection

**Figure 1:**
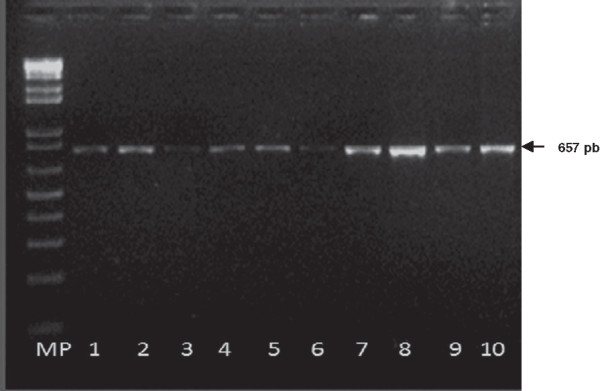
**Agarose gel showing the products of conventional PCR amplification of PCV2 from serum samples.** MP: molecular weight standards (100 bp ladder). Lane 1 – 9 are field positive samples; lane 10: positive control.

The results of the amplification of the PCV2 in serum samples tested by conventional PCR are illustrated in Figure 1. A total of 21 out of 110 whole serum samples (19.1%) collected during 2002 - 2005 were positive to PCV2. Those samples were from 13 farms located in Cundinamarca (6), Risaralda (4) and Valle (3). The two oldest positive samples detected in farms from Valle and Risaralda were from 2002 (Table 1). Additionally, PCV2 DNA was identified in 11 archived necropsy samples collected during PCVAD outbreaks that occurred in Colombia during 2006-2007. On the other hand, from the samples collected over the period of 2009 – 2010, PCV2 DNA was identified in 34 serum samples (25.2%) from 14 farms (51.8%) and in 40 tissue samples (80%) from 50 pigs that showed clinical signs compatible with PCV2-associated systemic infection (Table 2). It is important to mention that PCV2 antigen was demonstrated previously by IHC in the lymph nodes of these 40 PCR positive samples. Detection was also associated with histological changes suggestive of PCV2-associated systemic infection (granulomatous inflammation and lymphoid depletion) [23].

**Table 1:** **PCV2 DNA detection in serum samples and tissue samples collected during 2002-2007**

Year	Conventional PCR		
	Positive farms*	Positive serum	Positive tissue
		samples**	samples**
2002	2/2	2/10	
2003	3/6	4/30	
2004	4/9	6/45	
2005	4/5	9/25	
2006			6/6
2007			5/5
	13/22 (59.1%)	21/110 (19.1%)	11/11 (100%)

*Positive farms/total examined.

**Positive samples/total examined.

**Table 2:** **PCV2 DNA detection in serum samples and cases collected during 2009-2010**

	Conventional PCR			
	Serum samples		Tissue samples	
Geopraphic origin	Positive*	Positive**	Positive*	Positive**
	Farms	serum	Farms	tissue
Antioquia	7/10	20/50	6/6	15/15
Cundinamarca	2/7	4/35	6/6	12/21
Risaralda	3/5	3/25	1/2	3/4
Valle	2/5	7/25	4/4	10/10
	14/27	34/135	17/18	40/50

*Positive farms/total examined.

**Positive samples/total examined.

### PCV2 PCR specificity and sensitivity

There was no amplification with the designed primers for PCV2 ORF2 for any of the four bacterial or the five viral nucleic acids from swine pathogens used to test the specificity of the PCR under the conditions used in this study. The conventional PCR test detected PCV-2 DNA up to a dilution of 10^−3^, corresponding to 2.2 × 10^3^ TCID50/ml.

### Genetic characterization

**Figure 2:**
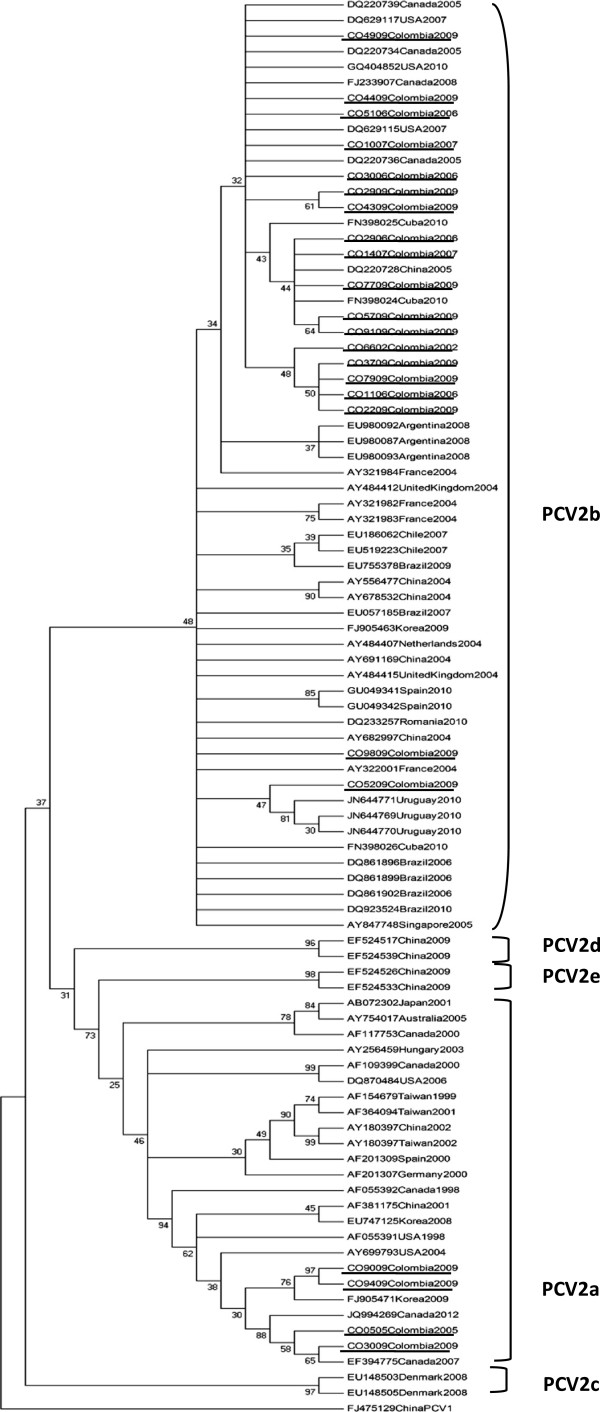
**Phylogenetic tree constructed by the neighbor-joining method based on the complete 702-nt sequence of the PCV2 ORF2. PCV2 Colombian strains evaluated in this study are shown underlined.** Sixty eight strains whose entire or partial sequences have been reported were included for comparison. PCV1 isolate FJ475129 was included as outgroup.

The complete genome of 23 Colombian ORF2 PCV2 sequences showed a length of 702 bp and revealed a nucleotide identity of 91.4% -100% among them. At the nucleotide level, the PCV2a and PCV2b sequences shared between them an identity of 98% -100% and 98.8 -100% respectively. The two genotypes of the PCV2 were found both in the strains isolated from PCV2-associated systemic infection affected animals and in those from healthy pigs belonging to the main pig-producing provinces of Colombia. The sequence analysis showed that PCV2b is the predominant genotype in Colombia (Figure 2). Interestingly, in two farms (farms 2 and 7), both genotypes, PCV2a and PCV2b, were detected before the first outbreak and they were also discerned during and after outbreaks of PCVAD in Colombia (Table 3).

**Table 3:** **Origin of 23 Colombian PCV2 ORF2 sequences examined in this study**

Year of the colection	PCV2 strain ID/original name	Geographic origin	Farm No.	Designation clinical condition	Sample origin	Genotype
2002	CO6602	Valle	7	Non Wasting	serum	PCV-2b
2005	CO0505	Cundinamarca	2	non wasting	serum	PCV-2a
2006	CO1106	Cundinamarca	2	Wasting	lymphoid node	PCV-2b
2006	CO2906	Valle	7	Wasting	lymphoid node	PCV-2b
2006	CO3006	Valle	18	Wasting	lymphoid node	PCV-2b
2006	CO5106	Valle	10	Wasting	lymphoid node	PCV-2b
2007	CO1007	Cundinamarca	6	Wasting	lymphoid node	PCV-2b
2007	CO1407	Antioquia	13	Wasting	lymphoid node	PCV-2b
2009	C02239	Antioquia	22	Wasting	lymphoid node	PCV-2b
2009	CO2909	Valle	8	non wasting	serum	PCV-2b
2009	CO3009	Valle	21	non wasting	serum	PCV-2a
2009	CO3739	Valle	37	Wasting	lymphoid node	PCV-2b
2009	CO4309	Valle	8	Wasting	lymphoid node	PCV-2b
2009	CO4409	Antioquia	12	non wasting	serum	PCV-2b
2009	CO4909	Cundinamarca	1	Wasting	lymphoid node	PCV-2b
2009	CO5209	Antioquia	29	Wasting	lymphoid node	PCV-2b
2009	CO5709	Antioquia	26	Wasting	lymphoid node	PCV-2b
2009	CO7709	Antioquia	5	Wasting	lymphoid node	PCV-2b
2009	CO7909	Antioquia	28	Wasting	lymphoid node	PCV-2b
2009	CO9009	Valle	18	Wasting	lymphoid node	PCV-2a
2009	CO9109	Antioquia	29	non wasting	serum	PCV-2b
2009	CO9409	Valle	18	Wasting	lymphoid node	PCV-2a
2009	CO9809	Risaralda	20	non wasting	serum	PCV-2b

**Figure 3:**
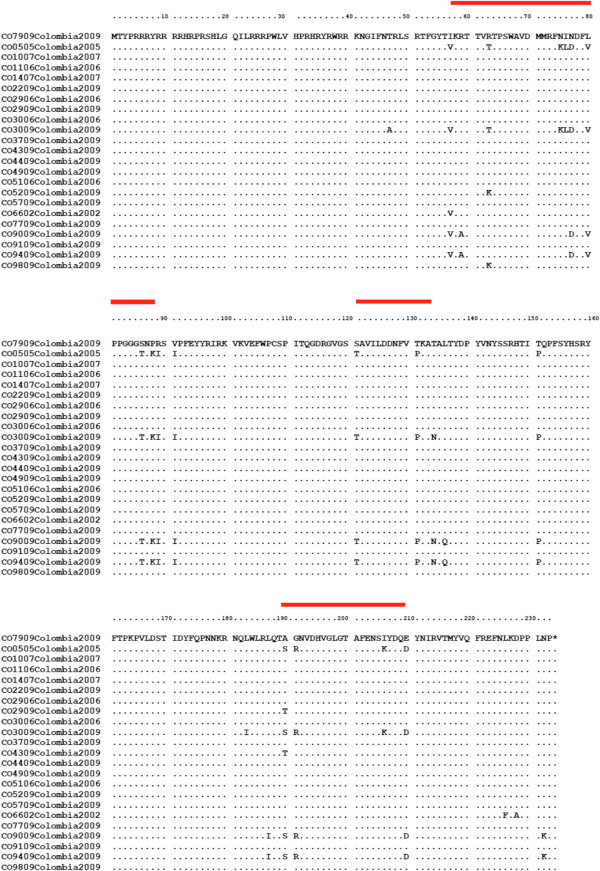
**Alignment of amino acids predicted from the ORF2 nucleotide sequences of the 23 Colombian PCV2 sequences during 2002-2010.** Amino acid changes are located between amino acid positions 57-89, 121-134 and 190-210 (highlighted in red lines) comparison. PCV1 isolate FJ475129 was included as outgroup.

The PCV2b Group included 19 strains that contained the signature motif CCCCGC encoding proline (P) and arginine (R) at nucleotides 262–267 and amino acids 88–89. Fifteen nucleotide substitutions that originated five amino acid substitutions: I57V, R63K, A190T, L226F and D228A were observed in the ORF2 from the PCV2b Colombian strains. On the other hand, the PCV2a Group consisted of four strains with the nucleotide sequence AAAATC encoding lysine (K) and isoleucine (I). Sixteen nucleotide substitutions were observed in the ORF2 from the PCV2a Colombian isolates, which led to twelve amino acid substitutions: T47A, R59A, T63R, K75N, L76I, T134N, L136Q, L183I, L187I, K206I and N232K. In this study the main positions of amino acid replacements among Colombian ORF2 PCV2 sequences were located at amino acid positions 57-89, 121–134 and 190-210 (Figure 3).

### Phylogenetic analyses

The phylogenetic analysis of the 23 ORF2 PCV2 sequences from this study along with 68 sequences published in the GenBank database that are representative of all PCV2 genotypes is shown in Figure 2. The phylogenetic tree reproduces all the PCV2 clusters previously described [11]. The 23 strains were divided into the two genotypes defined earlier, PCV2b (n = 19) and PCV2a (n = 4).

According to the nomenclature previously established [24] and supported by high confidence values, the ORF2 sequences from four Colombian PCV2a, were grouped with those described as the PCV2 emergent variants associated with severe porcine circovirosis reported in Canada (1998, 2007, 2012), USA (1998, 2004), China (2001), and Korea (2008, 2009) in a defined cluster within the genotype PCV2a (Figure 2). The remaining PCV2 sequence was clustered within the genotype PCV2b closely related to published sequences from Canada (2007, 2012), USA (2007 - 2010), as well as European, Asian, Caribbean and South American strains.

Sequence analysis and comparison of Colombian PCV2b sequences with strains reported from South America showed a very high level of homology. Identity of 98.1% -99.8% at the nucleotide level and 94.4% - 97.8% at the amino acid level was found when compared with six of the Brazilian strains included in this study (accession numbers DQ861896, DQ861899, DQ861902, EU057185, DQ923524 and EU755378). The comparison with Argentinean strains (EU980087, EU980092 and EU980093) revealed an identity ranging from 99% to 99.8% and 98.7% to 100% at the nucleotide and amino acid level, respectively. At the same time, Colombian PCV2b strains showed a high nucleotide (98.4%-99.7%) and amino acid (94.4 %-97.8%) identity with three of the Uruguayan strains included in this study (JN644769, JN644770 and JN644771). By contrast, the comparison with two Chilean strains revealed identity ranging from 92% to 95.8% at the nucleotide and 90.5% to 95.2% at the amino acid level.

## Discussion

PVC2 was detected in Colombian pigs associated with a wide variety of clinical conditions as described previously [22, 23]. In this study, the presence of PCV2 was demonstrated by PCR in 22.4% of the serum and 83.6% of the tissue samples examined. The identification of PCV2 not only in cases associated with PMWS but also in healthy pigs, suggests that various risk factors may contribute to the exacerbation of PCV2 infection and the development of associated lesions. An epidemiological survey of PCVAD conducted between November 2008 and December 2009 in Colombia identified some factors associated with high mortality rates in the three major swine rearing areas in the country [22, 23]. Herds that did not have good management practice, presence of poliserositis in weaned pig and low feed intake resulted in a major risk of increased mortality. In that study it was found that vaccination against PCV2, authorized since 2008, represented a high effective intervention practice on controlling PCAVD outbreak even though PCV2 infection itself wasn’t considered a risk factor for PCVAD development. It remains unclear which factors contributed to maintain a subclinical PCV2 infection in Colombian swine herds and thus it needs to be further investigated.

Unfortunately, samples collected before 2002 were not available at the time of the study, but it is reasonable to assume that PCV2 was already present in the Colombian pig population prior to this time frame. The earliest confirmed detection of PCV2 worldwide was in 1962 in Germany [25], before that, the virus was probably causing subclinical infections or remained unknown for many decades before the description of PCVAD as a disease complex. An alternative to gain access to the knowledge of the PCV2 infection is the use of archived material, it is how in the UK investigations in archived formalin-fixed material established PCV2 detection using TaqMan1-PCR and immunohistochemistry in material originated from the 1970s [26]. The earliest PCV2 infection in Swiss archived material was found in 1986 by using immunohistochemistry, resulting in the recognition of the earliest histological lesions typical for PCV2-associated systemic infection [27]. Similarly, a study from Spain revealed the presence of PCV2 in archived material from 1985 onwards, and the occurrence of typical PCV2-associated systemic infection lesions as early as 1986 [28]. A retrospective study of PCV2 infection in Japan reported seven cases in 1989 [29]. Also, the earliest PCV2 infection in Thailand was reported in 1993 by using Nested PCR from formalin fixed tissues of PCV2-associated systemic infection affected pigs [30].

When considering Colombia, the data presented here showed an increase in the incidence of PCV2b infection between 2006-2007, in that period PCVAD was epizootic and caused problems in numerous farms in several provinces [22]. During that time, PCV2 positive samples by immunohistochemistry were collected from pigs showing characteristic lesions of PCV2-associated systemic infection and some of them were later studied by PCR. As mentioned earlier, 50 cases were collected from pigs with wasting problems during a time period between 2009 -2010. Forty of these samples were PCR positive and PCV2 DNA was identified in the 25.2% of the serum samples collected at that time from healthy pigs. It is well known that detection of PCV2 alone, without the three criteria for diagnostic of PCVAD, does not indicate PCVAD but merely PCV2 infection [3]. However, there was no evidence that could relate the PCV2 strain groups and pathogenic PCV2 isolates from PCV2-associated systemic infection cases and besides that it cannot be concluded that PCV2 isolates from healthy pigs are non-pathogenic.

This study characterized and reconstructed phylogenetic analysis of 23 ORF2 of PCV2 strains obtained from pigs with PCV2-associated systemic infection and healthy pigs during 2002 -2010. Molecular characterization of the isolates was based on the analysis of cap gene. This region is suitable for genotyping studies and is considered a reliable phylogenetic marker for PCV2 strains since it is possible to reconstruct the same tree as with the whole viral genome [11]. Porcine circovirus type 2 (PCV2) is divided into two major genotypes based on sequencing analysis. Recently, both genotypes were proposed and referred to as PCV2a and PCV2b [31].

The alignment of the amino acid sequences of the ORF2 PCV2 capsid protein performed in the present study has identified three major regions of amino acid heterogeneity located at amino acid positions 57 -89, 121 – 134 and 190- 210 within heterogenic regions (Figure 3) similar to previous reports [24, 32]. It is interesting to note that two of these regions (57-89 and 121-134) correspond with two dominant immunoreactive areas (65-87 and 113-139) as identified by Pepscan analysis [32]. These immunodominant regions of the capsid protein of PCV2 exposed to selective immune pressure could represent potential candidate regions involved in the emergence of PCV2 variants. However, no repeatable or characteristic amino acid motifs for these two regions of the capsid protein of PCV2 could be associated with strains identified from pigs with PCV2-associated systemic infection or healthy pigs. Whether the anti-PCV2 antiserum generated from Colombian PCV2 strains could recognize the same epitopes in strains from other countries is not yet known but the results presented here contribute to the knowledge of the variability of the immunoreactive regions among PCV2 strains.

In terms of PCV2 genotype and its dynamics over time, there was not a relationship between the genotype of PCV2 and year of detection. Among the 23 Colombian PCV2 strains in this study, the strain CO6602 collected in 2002 belonged to the genotype PCV2b, whereas in the period from 2002 to 2010, in spite of being genotype 2b more prevalent, the isolates were a mix of genotypes PCV2a and PCV2b. The results presented here suggest that PCV2b has become the main genotype acting in Colombia over time. Previous studies revealed that both genotypes were associated with PCVAD-affected and non-affected herds [24, 33–35]. Nevertheless, PCV2b is currently prevailing in naturally occurring infections worldwide [36], a similar situation could be occurring in Colombian pig populations.

Furthermore, several recent publications have reported a shift from the genotype PCV2a to PCV2b which might be related to the occurrence of PCVAD outbreaks in Canada [37], Sweden [38], Switzerland [35] and Spain [39], indicating that PCV2b may be more virulent than PCV2a. However, in Colombia PCV2b has been present since 2002 in healthy animals and then it was associated to the PCVAD epizootic occurrence in farms of several regions of the country in the period 2006 – 2009. Nevertheless, in farm 2 a shift from PCV2a (2005) to PCV2b (2006) was found and, in farm 18 the variation was from PCV2b (2006) to PCV2a (2009). In addition, the PCV2b strains CO6602 and CO2906 collected in the same farm in the years 2002 and 2006 respectively showed less than 1.3% differences in the amino acid sequence of ORF2. It is well known that Colombia keeps a wide commercial exchange with North American countries, which includes the import of live animals and semen, so it is not surprising that the strains analyzed in this work were found to be closely related to Canadian and American strains isolated between 2004 - 2010, sharing 74,7% - 100% identity at the amino acid level. This coincides with findings in some countries, where the presence of PCV2 has been linked to imported pigs [40] and the movement of asymptomatic PCV2- infected pigs that occurs as a result of the swine trading which has been suggested to be responsible for the rapid spread of PCV2 around the globe. Unfortunately, there is a lack of information regarding the origin of breeding animals in the herds which limits the capacity to shed light over the potential source of infection and why it is not possible to determine the exact year of introduction of PCV2 in Colombian swine farms.

## Conclusions

In Colombia, through the results presented here, it has been demonstrated that PCV-2 is associated with PCVAD. In this study, 23 ORF2 strains of PCV-2 were obtained from animals with confirmed diagnosis of systemic and subclinical infection originated from different farms in a time frame from 2002 to 2010. Although in South America data regarding molecular characterization of PCV2 strains is still scarce, based on genotyping studies it has been concluded that PCV2b is the predominant circulating genotype in the region. However in Colombia there was a mixture of both genotypes during the epizootic, but recently PCV2b became more common in cases of PCVAD. This finding contributes to the understanding of the molecular epidemiology of PCV2 in Latin-American countries and may also help to establish the bases necessary to study the emergence of new viral variants in this region.

## Methods

### Samples

This study analyzed three different groups of samples, each one from a different time frame. The first group corresponded to a retrospective study that included a total of 110 blood serum samples belonging to the bank sera of the Instituto Colombiano Agropecuario (ICA) collected during 2002 - 2005 as part of the national swine serologic monitoring program which focused on farms with 50 or more sows. Serum samples were from nursery/grower pigs (6-12 weeks of age) from 22 previously reported seropositive farms and they were mostly from animals with unknown or healthy clinical status [22]. The second group of samples was from archived necropsy material collected between July 2006 and May 2007 from pigs with historical records of PCV2-associated systemic infection (by immunohistochemistry assays) from eleven different herds of four Colombian geographic regions (Table 1).

Additionally, a third group of serum and tissue samples collected between January 2009 and February 2010 were also examined (Table 2). Five pigs per farm from the 27 farms evaluated were used for serum collection. The criteria for serum selection included the age (between 8 and 15 weeks), weight (under 60 kg) and no clinical evidence of PCVAD in the animals. A total of 50 pigs, between 6 to 16 weeks old, with wasting problems after weaning were investigated in 18 of these farms. Field veterinarians selected 1 - 5 pigs from each farm based on loss of body condition, with the additional clinical signs of diarrhea, skin pallor, and/or respiratory disorders. Clinical samples (popliteal and inguinal lymph nodes, tonsil, spleen and kidney) of the affected euthanized animals were collected. The samples were kept at -70°C until performing DNA extraction and PCR analysis.

### DNA extraction and PCV2 DNA amplification

The DNA was extracted from 200μl of serum or 20 mg of organ tissue homogenate using a commercial kit (QIAamp DNA Mini Kit, Qiagen, USA) according to the manufacturer’s recommendations. To avoid cross contamination, samples were processed individually and stored at -20°C.

Conventional PCR for PCV-2 was performed using primers previously described [41] which amplified a 657 base pair (bp) fragment. The forward primer (5′-GCCAGTTCGTCACCCTTTC-3′) was located between genomic positions 940 and 958 (found in PCV2 ORF1). The reverse primer (5′-CTCCCGCACCTTCGGATAT-3′) was located between positions 1578 and 1596 (found in PCV2 ORF2). The optimized PCR reaction mixture contained 200 nM dNTPs, 1.5 mM MgCl, 1× PCR buffer, 500 nM of each primer and 0.05U of Taq polymerase (Promega M8298) in a 25 μl final volume.

The reactions were run in a thermocycler Bio Rad ALS-1296 (Bio-rad Laboratories, Inc USA) under the following conditions: one cycle at 94°C for 5 min, followed by 35 cycles of 94°C during 30 s, primer annealing 64°C for 1 min, initial extension at 72°C for 30 s, and a final extension of 72°C for 7 min. The amplified product was visualized by standard gel electrophoresis of 10 μl of the final reaction mixture on a 1.5% agarose gel (Sigma A-9539) in TBE Buffer 10× (Invitrogen 15581-044). Amplified DNA fragments of specific size were observed by ultraviolet fluorescence after staining with EZ Vision™ Three (Amresco N-313, USA). The length was verified by a 100 bp DNA ladder (Invitrogen 15628-019). Control DNA from a PCV2 strain (Genbank accession number JF290418) was included in each reaction.

### PCR amplification of ORF2 gene

One set of specific PCV2 primers, based on PCV2 genome from the strain ZhuJi2003 (AY579893) published in the Gen Bank was designed to amplify the complete ORF2 PCV2 sequence. A full-length ORF2 gene of PCV2 was amplified by PCR with forward primer (capFw 5′CCGTTGGAATGGTACTCCTC 3′) located between genomic positions 825 and 844 (found in PCV2 ORF1). The reverse primer (cap Rw 5′ ACAGCGCACTTCTTTCGTTT3′) was located between positions 1760 - 1741 (found in PCV2 ORF2). PCV2 specific primers amplified a 935 bp DNA fragment. The optimized PCR reaction mixture contained 200 nM dNTPs, 1.5 mM MgCl, 1× PCR buffer, 600nM of each primer and 0.05U Taq polymerase (Promega M8298). Reaction conditions were as follows: initial denaturation at 94°C for 5 min, followed by 35 cycles of 95°C during 45 s, primer annealing 57°C for 45 s, initial extension at 72° for 45 s, and a final extension of 72°C for 12 min.

The specificity of the primers was tested by adding extracted nucleic acids from several viral and bacterial swine pathogens such as: *Actinobacillus pleuropneumoniae, (*ATCC 27088*), Mycoplasma hyopneumoniae* (field strain), *Haemophilus parasuis* (ATCC 19417*), Streptococcus suis (*ATCC 700794), swine influenza virus H1N1 (A/SW/Iowa/H1N1 NVSL 003 IDV 9501), porcine reproductive and respiratory syndrome virus (NVSL 130 PDV 9801), Aujeszky’s disease virus (Shope strain NVSL 070 - PDV), porcine parvovirus (Mengeling strain NVSL 080- PDV9501) and porcine circovirus type 1. Control DNA from PCV1 was obtained from the supernatant of the PK15 cell line, which is persistently infected with this virus (ATCC CCL-33). The sensitivity of the PCV2 PCR was estimated through the evaluation of serial DNA dilutions extracted from the PCV2 positive control. The amplified products were run in a 1.5% agarose gel and visualized by staining with EZ VisionTM (Amresco N -313, USA).

### Viral sequences and phylogenetic analysis

DNA fragments of the calculated sizes were excised and recovered from the agarose gel using spin columns as described by the manufacturer (QIAquick Gel Extraction Kit, Qiagen 2876, USA). The purified PCR products were used as templates in cycle sequencing reactions primed with the PCV2 primers (capFw, capRw) and sequenced in both directions by Macrogen Sequencing Service, USA. Sequence alignment was performed using ClustalW software; genotype studies were performed by analyzing ORF2 compared to published sequences corresponding to different genotypes. The degree of identity among sequences at the nucleotide and amino acid levels was determined using BioEdit package v.7.0.9 [42]. The phylogenetic tree was constructed by neighbor-joining method with the Kimura two-parameter as the model of nucleotide substitution using MEGA v.5.0 software [43]. Confidence in the NJ tree was estimated by 1000 bootstrap replicates.

The sequencing of ORF2 presented greater difficulties with the electropherograms, with lower quality peaks. Only 23 were considered of good quality, with a definitive interpretation of the base sequence. The phylogenetic tree was constructed for ORF2 by comparing positive samples from various regions of Colombia as well as 68 sequences from the GenBank database, representative of all PCV2 genotypes described in North America, South America, Europe, Cuba and Asian countries (Table 4). The tree was rooted with a PCV1 sequence (accession number FJ475129).

**Table 4:** **Accession numbers and geographic origin of the ORF2 sequences included in the phylogenetic analysis**

GenBank accession	PCV2 strain ID/original	Country origin	Genotype	Genotype references
AB072302	No. 26	Japan	PCV-2a	Imai et al. (2001)
AF055391	nd	USA	PCV-2a	Meehan et al. (1998)
AF055392	nd	Canada	PCV-2a	Meehan et al. (1998)
AF109399	2-E	Canada	PCV-2a	Hamel et al. (2000)
AF154679	nd	Taiwan	PCV2-2a / 2B	Kuo et al. (1999)
AF117753	2-D	Canada	PCV-2a	Hamel et al. (2000)
AF201307	GER3	Germany	PCV-2a / 2C	Mankertz et al. (2000)
AF201309	SPA2	Spain	PCV-2a	Mankertz et al. (2000)
AF364094	nd	Taiwan	PCV-2a	Wang et al. (2001)
AF381175	BF	China	PCV-2a	Lu et al. (2001)
AY180397	Pingtung-5	China	PCV-2a	Liao et al. (2002)
AY256459	336	Hungary	PCV2-2a 2C	Dan et al. (2003)
AY321982	Fh14	France	PCV-2b	de Boisseson et al. (2004)
AY321983	Fh20	France	PCV-2b	de Boisseson et al. (2004)
AY321984	Fd3	France	PCV-2b	de Boisseson et al. (2004)
AY322001	Fh21	France	PCV-2b	de Boisseson et al. (2004)
AY484407	NL_Control_1	Netherlands	PCV-2b	Grierson et al. (2004)
AY484412	NL_Control_6	United Kingdom	PCV-2b	Grierson et al. (2004)
AY484415	NL_PMWS_3	United Kingdom	PCV-2b	Grierson et al. (2004)
AY556477	HuNan	China	PCV2- 2b 1C	Zhixin et al. (2004)
AY678532	ZS0401	China	PCV-2b	Zhou et al. (2004)
AY682997	ZC	China	PCV-2b	Wang et al. (2004)
AY691169	QZ0401	China	PCV-2b	Zhou et al. (2004)
AY699793	nd	USA	PCV2-2a 2E	Fenaux et al. (2004)
AY847748	BJW	Singapore	PCV2-2b 1B	Liu et al. (2005)
AY754017	Aust 6	Australia	PCV2-2a 2A	Muhling et al. (2005)
DQ151643	GS	China	PCV-2b	Ma et al. (2005)
DQ220728	FMV05-6317	Canada	PCV-2b	Tremblay et al. (2005)
DQ220734	FMV05-7389	Canada	PCV-2b	Tremblay et al. (2005)
DQ220736	FMV05-7537	Canada	PCV-2b	Tremblay et al. (2005)
DQ220739	FMV05-6302	Canada	PCV-2b	Tremblay et al. (2005)
DQ233257	ROM	Romania	PCV-2b	Cadar et al. (2007)
DQ629115	n32eu	USA	PCV-2b	Cheung et al. (2007)
DQ629117	k52	USA	PCV-2b	Cheung et al. (2007)
DQ861896	am22	Brazil	PCV-2b	Castro et al. (2006)
DQ861899	am9	Brazil	PCV-2b	Castro et al. (2006)
DQ861902	am21	Brazil	PCV-2b 1A	Castro et al. (2006)
DQ870484	hk102	USA	PCV-2a	Cheung et al. (2007)
DQ923524	15/23R	Brazil	PCV-2b	Dezen et al. (2010)
EF524517	GS04	China	PCV-2d	Wang et al. (2009)
EF524526	LN05	China	PCV-2e	Wang et al. (2009)
EF524533	GX0602	China	PCV-2e	Wang et al. (2009)
EF524539	TJ06	China	PCV-2d	Wang et al. (2009)
EU057185	P0404c/03	Brazil	PCV-2b	Esteves et al. (2007)
EU148503	DK1980PMWSfree	Denmark	PCV-2c	Dupont et al. (2008)
EU148505	DK1990PMWSfree	Denmark	PCV-2c	Dupont et al. (2008)
EU186062	Chile C	Chile	PCV-2b	Bucarey et al. (2007)
EF394775	05-22779	Canada	PCV2 –2a 2E	Tremblay et al. (2007)
EU519223	Chile-I	Chile	PCV-2b	Bucarey et al. (2007)
EU747125	PCU1	Korea	PCV-2a	Vijayachandran et al. (2008)
EU755378	BRA9	Brazil	PCV-2b	Chiarelli-Neto et al. (2009)
EU980087	isolate 5	Argentina	PCV-2b	Pereda et al. (2008)
EU980092	isolate 11	Argentina	PCV-2b	Pereda et al. (2008)
EU980093	isolate 11	Argentina	PCV-2b	Pereda et al. (2008)
FJ233907	SoPCV2b	Canada	PCV-2b	Chaiyakul et al. (2008)
FJ475129	BJ-1	China	PCV1	Zhou et al. (2008)
FJ905463	C7155	Korea	PCV-2b	Kim et al. (2009)
FJ905471	C7189	Korea	PCV-2a	Kim et al. (2009)
FN398024	Villa Clara V2	Cuba	PCV-2b	Pérez et al. (2010)
FN398025	Villa Clara V4	Cuba	PCV-2b	Pérez et al. (2010)
FN398026	Pinar del Rio	Cuba	PCV-2b	Pérez et al. (2010)
GQ404852	MN614	USA	PCV-2b	Li et al. (2010)
GU049341	Sp-10-7-54-13	Spain	PCV-2b	Fort et al. (2010)
GU049342	Sp-10-7-54-13	Spain	PCV-2b	Fort et al. (2010)
JN644769	SeUy1	Uruguay	PCV-2b	Ramos et al. (2010)
JN644770	SeUy2	Uruguay	PCV-2b	Ramos et al. (2010)
JN644771	SeUy3	Uruguay	PCV-2b	Ramos et al. (2010)

## Abbreviations

PCVAD: Porcine circovirus-associate disease
PCV2: Porcine circovirus type 2
DNA: Deoxyribonucleic acid
PCVD: Porcine circovirus diseases
ORF: Open reading frame
TCID50: 50% Tissue Culture Infective Dose
I: Isoleucine
V: Valine
K: Lysine
A: Alanine
T: Threonine
L: Leucine
F: Phenylalanine
D: Aspartic acid
K: Lysine
N: Asparagine
Q: Glutamine
PCR: Polymerase chain reaction
dNTPs: Deoxynucleotide triphosphates
MgCl: Magnesium chloride
ATCC: American Type Culture Collection
NVSL: National Veterinary Services Laboratories.
